# Guided Self-Assembly of Nano-Precipitates into Mesocrystals

**DOI:** 10.1038/srep16530

**Published:** 2015-11-12

**Authors:** H. Liu, Y. Gao, Z. Xu, Y.M. Zhu, Y. Wang, J.F. Nie

**Affiliations:** 1 Department of Materials Science and Engineering, Monash University, Clayton, Victoria 3800, Australia; 2 Department of Materials Science and Engineering, The Ohio State University, 2041 College Road, Columbus, OH 43210, USA

## Abstract

We show by a combination of computer simulation and experimental characterization guided self-assembly of coherent nano-precipitates into a mesocrystal having a honeycomb structure in bulk materials. The structure consists of different orientation variants of a product phase precipitated out of the parent phase by heterogeneous nucleation on a hexagonal dislocation network. The predicted honeycomb mesocrystal has been confirmed by experimental observations in an Mg-Y-Nd alloy. The structure and lattice parameters of the mesocrystal and the size of the nano-precipitates are readily tuneable, offering ample opportunities to tailor its properties for a wide range of technological applications.

Mesocrystals consisting of a periodic distribution of uniform nano-precipitates whose structure and composition are different from those of the matrix may have remarkable properties and functionalities ranging from photonic^1^,^2^,^3^, phononic^4^, thermoelectric^5^, optical^6^, to even mechanical^7^,^8^. These properties enable a broad range of advanced technological applications. Precise control of the periodicity and regularity of the spatial distribution of the nano-precipitates and of the uniformity of their sizes (ranging from nanometers to micrometers) are critical to achieve desired properties of mesocrystals. Several advanced techniques of synthesis have been employed in fabricating mesocrystals, including the bottom-up self-assembly using regular templates produced by either lithography^9^,^10^ or dislocation networks at hetero-phase interfaces between films and substrates^11^. Lithography is a rather expensive and time consuming technique with size limitations^6^. The nano-precipitates grown on the templates fabricated by lithography are often not easily scalable. The periodic strain fields associated with misfit dislocations at the hetero-phase interfaces have been utilized to guide the nucleation and growth of second phase particles^12^,^13^,^14^ into nearly regular patterns^11^,^15^. However, the type and distribution of dislocations at the hetero-phase interfaces are difficult to control in comparison to homo-phase interfaces (i.e., grain boundaries) and, thus, the distribution of the nano-islands is not easily tuneable.

In this study, we utilize a highly regular hexagonal dislocation network formed at a twisted grain boundary as a template to guide the self-assembly of multiple deformation (or correspondence) variants of a precipitate phase into a mesocrystal having a honeycomb structure. By applying crystallographic theory and phase field microelasticity theory of phase transformations and deformation in solids^16^,^17^, we first analyse the shape and spatial distribution of precipitates and their interaction with dislocations for possible guided self-assembly. Using the highly anisotropic β_1_ precipitates in Mg-Nd and Mg-Y-Nd alloys as examples, we show how the hexagonal dislocation network is utilized as a template to guide the formation of a honeycomb mesocrystal consisting of nano-precipitates of different variants of the β_1_ phase. Such mesocrystals may have exceptional properties for advanced functional and structural applications. The design method, *phase transformation pathway and defect structure engineering*, creates a new opportunity for the development of highly tuneable 3D mesocrystals of nano-precipitates in bulk materials.

## Results

**Figure 1:**
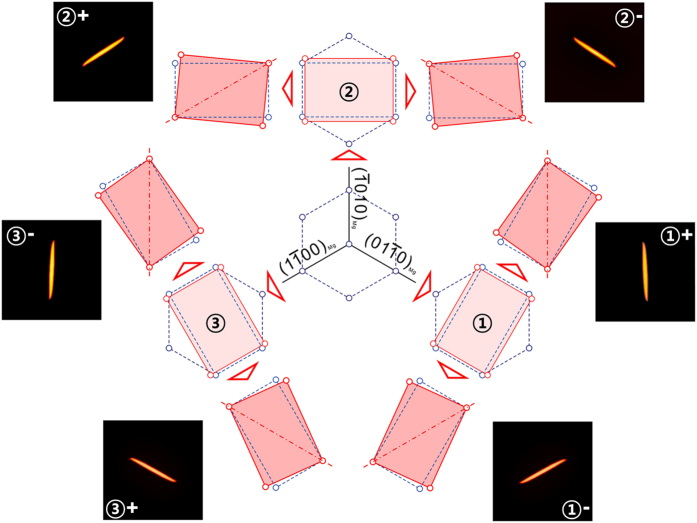
Schematic diagram showing the lattice transformation from α−Mg matrix to three deformation variants of β_1_ phase, and six orientation variants of β_1_ phase, and phase field simulation results of their morphologies.

**Figure 2:**
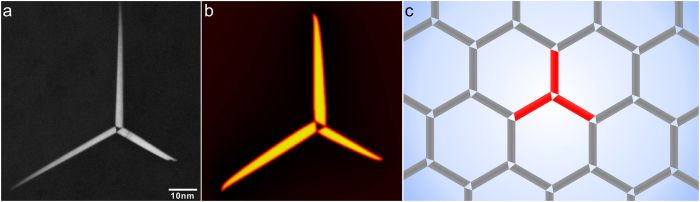
(**a**) HAADF-STEM image and (**b**) phase field simulation of a triadic structure of β_1_ particles in the Mg-3 wt% Nd alloy. (**c**) The triadic structure in (**a**,**b**) can act as the building block of a honeycomb structure.

The idea of the current work is inspired by a triadic assembly of three orientation variants of β_1_ precipitates in the α-Mg matrix frequently observed in Mg-Nd and Mg-Y-Nd alloys^19^,^20^,^21^,^22^. Each precipitate has a plate shape with 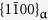 habit plane. This triadic structure originates from the breaking of the three-fold symmetry of the hexagonal close-packed (h.c.p.) matrix during the α−Μg to β_1_ transformation. According to the lattice correspondence proposed for the α−Μg (point group: *6/mmm*) to β_1_ (point group: 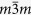) transformation^19^, i.e., 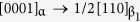, 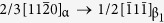, and 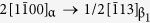, there are three equivalent transformation pathways, leading to three crystallographically equivalent correspondence variants of the β_1_ phase^18^,^19^,^20^,^21^,^22^. Each correspondence variant can generate two orientation variants by two opposite rigid-body rotations (clockwise or counter-clockwise) (Fig. 1). For example, the lattice of the correspondence variant ② can rotate clockwise or anticlockwise to form a plate with the (1–100) or (01–10) habit plane, leading to two orientation variants designated as variant ②+ or ②−. For a given plate, stress concentration exists near its end rims, which becomes the dominant driving force for self-assembly. If three such plates belonging to different correspondence variants can form in a triadic assembly (i.e., ①+/②+/③+ or ①−/②−/③−), then the stress concentrated at one end rim of each plate can be largely removed. Such a triadic assembly has been observed in both experiments^19^,^22^,^23^,^24^ (Fig. 2a) and computer simulations^20^,^21^ (Fig. 2b).

Such a stable triadic assembly provides the basic building block of an interconnected honeycomb structure (a hexagonal network of β_1_ precipitates, Fig. 2c). However, even with autocatalysis caused by the long-range elastic interactions, nucleation of β_1_ precipitates is a stochastic process and the triadic building blocks will not arrange themselves into a honeycomb structure without appropriate guidance. Thus, a template that not only lowers the activation energy barrier for β_1_ nucleation at desirable sites, but also selects a proper β_1_ variant for a given site (i.e., variant selection) is needed. We will show below that a hexagonal dislocation network consisting of three sets of screw dislocations (all right-handed or left-handed) is an ideal candidate template. This type of dislocation network is frequently observed on (0001) plane in h.c.p. crystals or on {111} planes in face-centred cubic (f.c.c.) crystals^24^,^25^,^26^,^27^,^28^,^29^,^30^,^31^,^32^,^33^, which is equivalent to a pure 〈0001〉 or 〈111〉 twisted grain boundary, respectively. The Burgers vectors of the three sets of dislocations in an h.c.p. crystal are 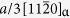, 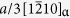 and 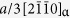, respectively, where *a* is the lattice constant of the α−Mg matrix.

**Figure 3:**
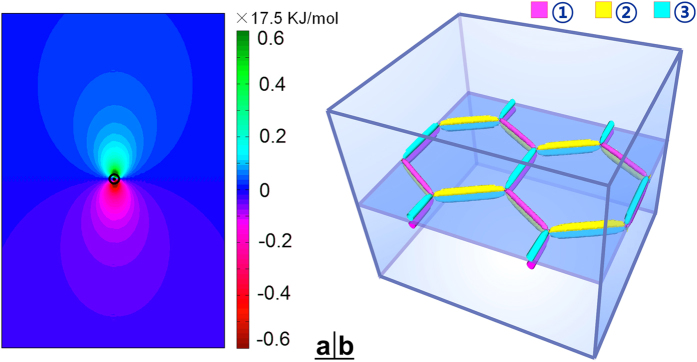
(**a**) Calculated values of interaction energy between the stress fields of a screw dislocation and a β_1_ variant ①+ that is to be nucleated near this dislocation. (**b**) Schematic plot showing energetically favoured nucleation sites for three deformation variants of β_1_ in regions immediately adjacent to the hexagonal dislocation network.

To explore whether such a dislocation network can bias nucleation and guide the self-assembly of β_1_ precipitates into a honeycomb structure, the interaction energies between the dislocation network and correspondence variants of β_1_ phase are firstly calculated. The interaction energy between a pre-existing screw dislocation, i.e., a line segment of the hexagonal dislocation network, and a to-be-nucleated β_1_ particle having a stress-free transformation strain (SFTS) of 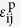
is given by^34^,^35^:


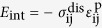


where 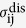 is the stress field around the dislocation and 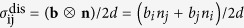, *b* and *n* are the Burgers vector of the dislocation and slip plane normal, respectively, and *d* is the inter-planar spacing of the slip plane^36^,^37^,^38^,^39^,^40^,^41^,^42^,^43^,^44^. The 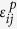 is the SFTS of the *p*-th correspondence variant of β_1_ (*p* = ①, ② and ③). A negative value of the interaction energy means that the formation of β_1_ under the influence of the stress field of the dislocation is energetically favoured. It is found that, within the basal plane containing the dislocation network, the interaction energies for all β_1_ variants are zero. For the plane lying one Burgers vector (a typical cut-off radius for dislocation core) above the segment of the dislocation network with 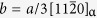, the interaction energies for the correspondence variants ①, ② and ③ are 10.15, 0.0 and -10.15 KJ/mol, respectively. These results mean that only variant ③ is favoured. Via further examination of the two orientation variants ③+ and ③− associated with the correspondence variant ③, we find that only variant ③− has its habit plane parallel to the pre-existing screw dislocation line and, thus, is the most favoured orientation variant. Similarly, for the plane lying below the dislocation-network-containing plane by one Burgers vector, the interaction energies for the three correspondence variants ①, ② and ③ are −10.15, 0.0 and 10.15 KJ/mol, respectively. In this case, the orientation variant ①+ becomes the most favoured one (Fig. 3a).

When a hexagonal screw dislocation network is introduced, the interaction energy calculation results suggest that the orientation variants ①−, ②− and ③− (represented by different colours) will form preferably on one side of the dislocation network in an alternating sequence, Fig. 3b, the growth of which will eventually lead to a mesocrystal having a honeycomb structure. Similarly, a mesocrystal of a honeycomb structure consisting of alternating distributed variants ①+, ②+ and ③+ will form on the other side of the dislocation network. The lattice parameter of the mesocrystal is determined by the size of the unit cell of the dislocation network that depends solely on the twist angle. The size of the mesocrystal is determined by the equilibrium volume fraction of the precipitate phase. According to Frank’s formula^25^,^28^, the size of the unit cell (i.e., the hexagon) in a dislocation network increases as the twist angle decreases. Thus, the maximum size of the hexagon is limited by the grain size of the α−Mg matrix, while the minimum size is dictated by the core width of the dislocation, which is determined by the interplay between the crystalline energy and the elastic strain energy^25^,^29^.

**Figure 4:**
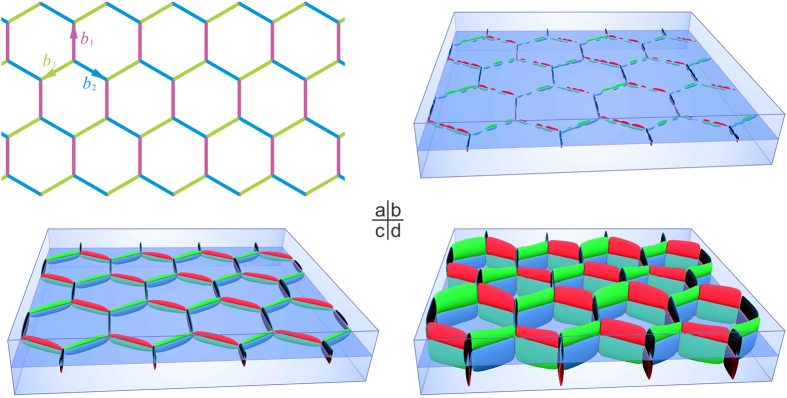
Schematic diagrams showing (**a**) a template of a hexagonal network of screw dislocations, (**b**–**d**) nucleation and growth of 6 variants of β_1_ precipitates on the template shown in (**a**). The Burgers vectors of orchid (*b*_*1*_), sky-blue (*b*_*2*_) and yellow-green (*b*_*3*_) segments in (**a**) are 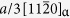, 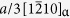 and 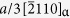 respectively. The orientation variants belonging to the three deformation variants ①, ②, ③ are represented by red, green and blue colors respectively.

To examine whether such a dislocation-guided self-assembly of β_1_ precipitates would lead to a mesocrystal of the honeycomb structure, heterogeneous nucleation and growth of β_1_ precipitates are simulated by the phase field method. By introducing the stress field of a dislocation network, as described above, the nucleation of β_1_ precipitates is simulated by using the Langevin random force term. In the simulations, the edge length of the hexagonal dislocation network is set to be 150 nm (corresponding to a misorientation angle of 0.01°), and the Burgers vector of each segment of the dislocation network is shown in Fig. 4a. In the early stages, isolated β_1_ particles nucleate on the dislocation network and grow along the dislocation line segments, Fig. 4b. Note that the precipitates on different sides of the plane containing the dislocation network cannot grow across the plane because it is elastically unfavourable. When all these particles impinge upon each other, a honeycomb structure of alternating β_1_ orientation variants forms, Fig. 4c. With prolonged ageing, the β_1_ precipitates grow mainly along the [0001]_α_ direction forming thin plates, until the equilibrium volume fraction of the β_1_ phase is reached, Fig. 4d.

**Figure 5:**
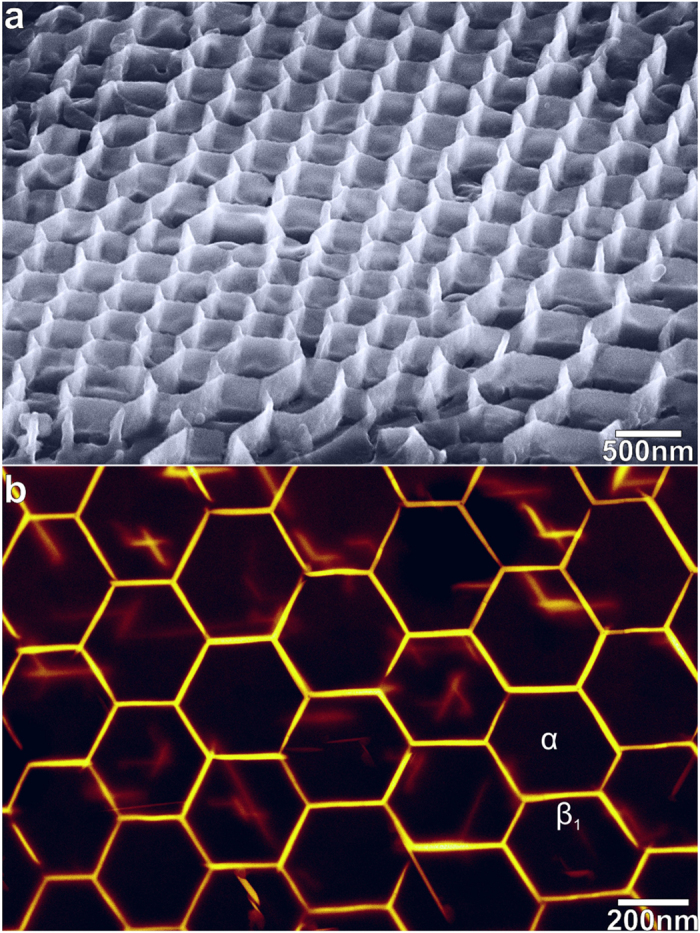
(**a**) Secondary electron SEM image and (**b**) HAADF-STEM image showing a honeycomb network of precipitates in the WE54 alloy. In this figure, β_1_ particles are bright since Nd has the higher atomic numbers than Mg.

The experimental support to the above design and simulation prediction is shown in Fig. 5. Figure 5a shows a honeycomb network of β_1_ plates in the WE54 alloy that has been solution treated for 8 hours at 525 °C (798 K), water quenched and aged for 10 hours at 250 °C (523 K). To facilitate the visualization, the sample was deeply etched to remove the α-Mg matrix inside each hexagon of the honeycomb structure. The hexagons in this honeycomb structure have edge length ranging from 150 to 300 nm. For each hexagon, each of its 6 faces is made by a single β_1_ plate, as shown more clearly by the high-angle annular dark-field scanning transmission electron microscopy image, Fig. 5b.

## Discussion

As a commonly observed physical phenomenon, symmetry breaking accompanying solid-state phase transformations provides a natural way to achieve self-accommodated domain structures. Depending on the final structure required, an appropriate phase transformation should be selected according to crystal symmetry and phase transformation mechanism. In our case, because three correspondence variants and three different habit planes are necessary to build the honeycomb structure, the h.c.p. to cubic phase transformation in the Mg-Y-Nd alloy system is selected. During the transformation, the (0001)_α_ plane transforms into 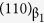 plane and thus the three-fold rotational symmetry of the h.c.p. structure is broken, leading to three crystallographically equivalent correspondence variants. Such a symmetry breaking generates the basic building block, i.e., the triadic assembly of three β_1_ variants, which guarantees the stability of the building block. This is essential for a honeycomb mesocrystal. Because of undergoing different transformation pathways and having different SFTS, the three β_1_ variants can respond distinctively to the stress fields of different dislocations, which governs the variant selection and self-assembly process. Such a selection originates from the correspondence between the dislocations and the β_1_ variants (e.g., the dislocation with the Burgers vector 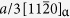 leads to variants ①+ and ②−), both of which are dictated by the breaking of the three-fold symmetry of the h.c.p. lattice. Since the eigenstrains of both precipitates and dislocations are related directly to the crystal structure, a feasible design strategy based on the phase transformation crystallography to produce self-assembled mesocrystals with various structures and lattice parameters can be proposed.

Comparing with existing methods of making regularly distributed second phase particles on substrates, the following distinctions of the method presented in this work should be noted:

- Both the lattice parameters and the structure of the mesocrystal can be tuned by adjusting dislocation type and spacing via controlling the type (e.g., tilt, twist, mixed) and misorientation of grain boundaries.
- The method can produce stacks of 2D mesocrystals or directly 3D mesocrystals by the utilization of 3D dislocation networks as templates. The resultant mesocrystal is easily scalable via standard physical metallurgy processes such as thermomechanical treatments. For example, pure twist and pure tilt grain boundaries can be created easily by polygonization of a twisted or bent single crystal upon annealing.
- The symmetry and motif of the mesocrystal could also be controlled by choosing appropriate phase transformations between different crystal structures in different alloy systems.
- The multiple variants in the honeycomb mesocrystal are dominated by symmetry breaking and long range elastic interactions, which provides distinctive symmetry characteristics comparing to the existing methods that produce mono-variant mesocrystals such as single variant f.c.c. (111) islands on f.c.c. (111) substrate.
- The interaction energy calculation results and phase field simulation results indicate that the nano- precipitates can form on both sides of the dislocation network, offering one more degree of freedom as compared to nano-island formation on substrates.
- Because the mesocrystal synthesized in this study is thermodynamically stable, the compositions of the precipitate and matrix phases are in a tie-line, there is no mixing or interdiffusion between the precipitates and matrix and thus the system developed could be applied at relatively high temperatures up to the solvus temperature.
- Since bulk samples are producible in this approach, one could explore mechanical properties in addition to the physical properties of these mesocrystals, which is rather difficult to do if using a thin film sample.

In summary, a new approach to develop mesocrystals is demonstrated through a combination of crystallographic design and defect engineering. The design concept and predicted mesocrystals have been confirmed by both computer simulation and experimental characterization. In the crystallographic design, symmetry breaking caused by a phase transformation is utilized, which provides a natural way to obtain fundamental building blocks for the mesocrytal. Furthermore, a hexagonal dislocation network is introduced to guide the self-assembly (via nucleation and growth) process of the precipitates leading to a honeycomb structure. Such a design method could provide a great deal of flexibility in synthesizing highly regular, highly tuneable nanostructures in bulk quantity, through *crystallographic design of phase transformation* and *defect engineering of dislocations*, and it is likely that many interesting mesocrystals could be produced.

## Methods

### Sample preparation

Plates of magnesium cast alloy WE54, with a nominal composition of Mg-(5.0–5.5)Y-(1.5–2.0)Nd-(1.5–2)HRE(heavy rare earth)-0.4Zr (wt%), were purchased from Magnesium Elektron Ltd. England. The dimensions of the plates are 1800 × 1800 × 25 mm. The as-received plates have already been solution treated at 525 °C for 8 hours, air cooled, and aged at 250 °C for 16 hours. They were grit blasted and pickled in 15% nitric acid before delivery. Small blocks with a dimension of 10 × 5 × 20 mm were cut from one plate and re-solution treated for 8 hours at 525 °C, water quenched and aged for 10 hours at 250 °C. The alloy with nominal composition of Mg-3 wt% Nd was cast from high purity Mg and Nd by induction melting in a steel mould crucible under a protected argon atmosphere at 760 °C, and pouring into a preheated mould coated by graphite. Bulk samples with dimensions of 8 mm × 11 mm × 20 mm were covered by MgO powder, solution treated at 520 °C for 24 hours, followed by water quenching and ageing for 10 hours at 250 °C.

### Scanning electron microscopy (SEM) and scanning transmission electron microscopy (STEM)

The SEM samples were prepared from heat treated blocks, which were first ground on SiC sand papers with increasing fineness grades up to 2400-grit and subsequently polished by 1 μm OPS suspension on Struers cloth. The samples were etched for about 10 seconds with a solution containing 29 ml picric acid, 41 ml water, 50 ml acetic acid and 350 ml ethanol. Secondary electron SEM images were recorded by a JSM-7001 F SEM with field emission gun working at 10 kV with a working distance of 15 mm and a probe current of 10 (~500 pA). For the preparation of TEM samples, small discs with a diameter of 3 mm were punched from strips cut from the heat treated blocks and manually ground to thicknesses around 150 μm. The final TEM foils were prepared by twin-jet electro-polishing at −55 °C and 0.1 A with a solution of 5.3 g lithium chloride, 11.16 g magnesium perchlorate, 500 ml methanol and 100 ml 2-butoxy-ethanol. The high-angle annular dark-field (HAADF) STEM images were acquired using a FEI Titan^3^ 80–300 operated at 300 kV, fitted with two CEOS aberration correctors, and a Fischione HAADF detector. In order to improve interpretability, the convergence angle was set to conservative 15.0 mrad, leading to a diffraction limited (Gaussian) probe diameter of ~0.12 nm.

### Phase field simulation

In the model system, face centred cubic β_1_ particles are precipitated out from the hexagonal α−Mg matrix under the influence of the stress field of a (0001)_α_ twist grain boundary. Model inputs, including lattice parameters, precipitate–matrix orientation relationship, elastic constants and free energy data, are obtained from experimental characterization, *ab initio* calculations and thermodynamic databases.

### Equipment and settings

The schematic diagrams in Figs. 1 and 2c were plotted by CorelDRAW Home & Student X7. Figures 2b, 3 and 4b,c,d were obtained according to phase field method and plotted by ParaView 3.8.0. The SEM image in Fig. 5a was processed using Image J 1.49B. The HAADF-STEM images in Figs. 2a and 5b were acquired using TEM image and analysis (TIA) software running in Titan. All the figures were combined and processed in CorelDRAW Home & Student X7.

## Additional Information

**How to cite this article**: Liu, H. *et al.* Guided Self-Assembly of Nano-Precipitates into Mesocrystals. *Sci. Rep.* **5**, 16530; doi: 10.1038/srep16530 (2015).
